# Travel restrictions during pandemics: A useful strategy?

**DOI:** 10.1063/5.0028091

**Published:** 2020-11-17

**Authors:** Massimiliano Zanin, David Papo

**Affiliations:** 1 Instituto de Física Interdisciplinar y Sistemas Complejos (IFISC) (CSIC-UIB), Campus UIB, 07122 Palma de Mallorca, Spain; 2 Center for Translational Neurophysiology for Speech and Communication, Fondazione Istituto Italiano di Tecnologia, via Fossato di Mortara 17/19, 44121 Ferrara, Italy

## Abstract

Though carrying considerable economic and societal costs, restricting individuals’ traveling freedom appears as a logical way to curb the spreading of an epidemic. However, whether, under what conditions, and to what extent travel restrictions actually exert a mitigating effect on epidemic spreading are poorly understood issues. Recent studies have actually suggested the opposite, i.e., that allowing some movements can hinder the propagation of a disease. Here, we explore this topic by modeling the spreading of a generic contagious disease where susceptible, infected, or recovered point-wise individuals are uncorrelated random-walkers evolving within a space comprising two equally sized separated compartments. We evaluate the spreading process under different separation conditions between the two spatial compartments and a forced relocation schedule. Our results confirm that, under certain conditions, allowing individuals to move from regions of high to low infection rates may turn out to have a positive effect on aggregate; such positive effect is nevertheless reduced if a directional flow is allowed. This highlights the importance of considering travel restriction policies alternative to classical ones.

At the beginning of any major pandemic, i.e., when cases are still restricted to one or few countries, governments worldwide try to prevent the disease from entering their territories by limiting international travels. The same strategy is also applied at a more local scale, e.g., by restricting travels between regions, also after the disease has reached all of them. It is nevertheless easy to see that an individual living in a region with a high infection rate would benefit from moving to a lower rate one; the question then becomes whether and under which conditions are those restrictions really beneficial. Using a simple random-walker model, we here show that travel restrictions are beneficial for regions with low infection rates, as indeed new imported cases are limited; nevertheless, this goes against the interest of the system as a whole, as free movements can reduce the total number of cases.

But after all that was or could be done in these Cases, the shutting up of Houses, so as to confine those that were well with those that were sick, had very great inconveniences in it, and some that were very tragical, and which merited to have been consider’d if there had been room for it; but it was authoriz’d by a law, in had the publick Good in view, and the end chiefly aim’d at, and all the private injuries that were done by putting it in execution must be put to the account of the publick benefit. It is doubtful to this day, whether in the whole it contributed any thing to the stop of the infection, and indeed, I cannot say it did.

Daniel Defoe, *History of the Plague in England*, 1722.^1^

## I. INTRODUCTION

In the event of the emergence of a new influenza virus strand, or more generally of a new contagious condition, governments worldwide try to reduce its impact through the adoption of different containment strategies. Among the few available nonpharmaceutical measures, one of the most common is the application of travel restrictions, e.g., closing international (or even local) borders. The rationale for this measure is easy to see: if no cases are reported in a given territory, isolating it from the outside, i.e., allowing no one from entering it, must necessarily block importing the disease. The ongoing coronavirus disease 2019 (COVID-19) pandemics,^2^ caused by the new severe acute respiratory syndrome coronavirus 2 (SARS-CoV-2), is no exception: countries closed borders with the exception of essential travels, most international flights were canceled, and some countries (e.g., Italy, Spain, and US) even imposed restrictions to inter-regional travels.^3–10^

Travel restriction policies are nevertheless at odds with some theoretical and numerical evidence suggesting that such policies may not always reduce spreading. Specifically, their effectiveness requires two important pre-requisites: (i) no cases should be present in the isolated territory, something not easy to guarantee especially when dealing with asymptomatic cases and (ii) travel bans should be total to avoid cross-territory propagation.^11^ In more real scenarios, travel restrictions have been shown to have a limited value, contributing at best to delay the introduction of the disease in a region.^12–15^ Nevertheless, if those restrictions are not complemented with local containment policies, they may even increase severity.^13^ Numerical simulations have shown that such policies could have a major impact only in the case of small islands;^16^ in the case of COVID-19, simpler strategies such as hand washing, self-isolation, and household quarantine may be more effective than travel restrictions.^3^ Additionally, *a posteriori* data analysis indicated that travel restrictions had a limited impact when compared with, e.g., complete lockdowns.^9,10^ Consequently, the World Health Organization recommended not to apply travel or trade restrictions to countries that are experiencing COVID-19 outbreaks.^5^

One common assumption behind all previously discussed modeling efforts is that travel restrictions are imposed at the beginning of the spreading process when the distribution of cases is highly asymmetric. In the prototypical case, one assumes that a new disease has emerged in a given country; the effort is then to avoid its propagation to all other countries, which are still disease-free. Nevertheless, the recent case of COVID-19 has shown that governments tend to keep these policies in place for a long time in order to isolate themselves from territories with a higher incidence rate.

Recently, a series of publications^17–20^ has explored an intriguing alternative: lifting late travel restrictions could actually help reducing the global incidence of the pandemic. The rationale behind this idea can be easily described through a thought experiment. Suppose there are two regions A and B, respectively, a large and highly populated city and a rural area. *Ceteris paribus* (i.e., the same virus strand and, therefore, same infectivity per contact), a higher incidence rate is to be expected in A, as a higher people density and a smaller inter-person distance will facilitate the propagation. A healthy person in A would thus have a higher probability of contracting the disease and would benefit from moving to B. Similarly, a diseased person will likely infect more people in A as compared to in B; hence, while from B’s perspective, that person ought to remain in A, from a global point of view, it is advantageous to relocate him/her to B.

While the original work focused on recurrent mobility,^17–20^ i.e., when people periodically commute between places of work and residence, we here explore whether the same effect is observed for unidirectional travels. Specifically, we suppose that people try to leave the city (and its high number of cases) to go to a second residence in a rural area, with the idea of staying there until the situation gets under control. This is motivated by the real behavior observed in Spain during the COVID-19 pandemics and specifically by a movement from Madrid to small coastal towns.^21,22^ Additionally, the authors of Refs. 17–20 resorted to a multiplex metapopulation model, which presents the advantage of being able to simulate heterogeneous populations—e.g., people of different socioeconomic classes. On the other hand, we here opt for a simpler but easier to understand model, based on a spatial SIR (Susceptible, Infectious, Recovered) dynamics^23,24^ with mechanistic people movements,^25^ in which each person moves through an uncorrelated random walk.

Consistent with previous results, we show that allowing free movement of people from high- to low-density regions is beneficial for the system as a whole although at the cost of a higher incidence rate in the low-density area. This latter cost can nevertheless be mitigated by performing tests at the border, by allowing only healthy people to relocate, and by limiting the mobility of relocated people. Additionally, the global benefit is reduced if people are allowed to return to their main residence; the key to containing pandemics thus resides in unidirectional flows. We finally discuss the implication and limitations of these results for health policies.

## II. INITIAL PROPAGATION MODEL

**FIG. 1.**
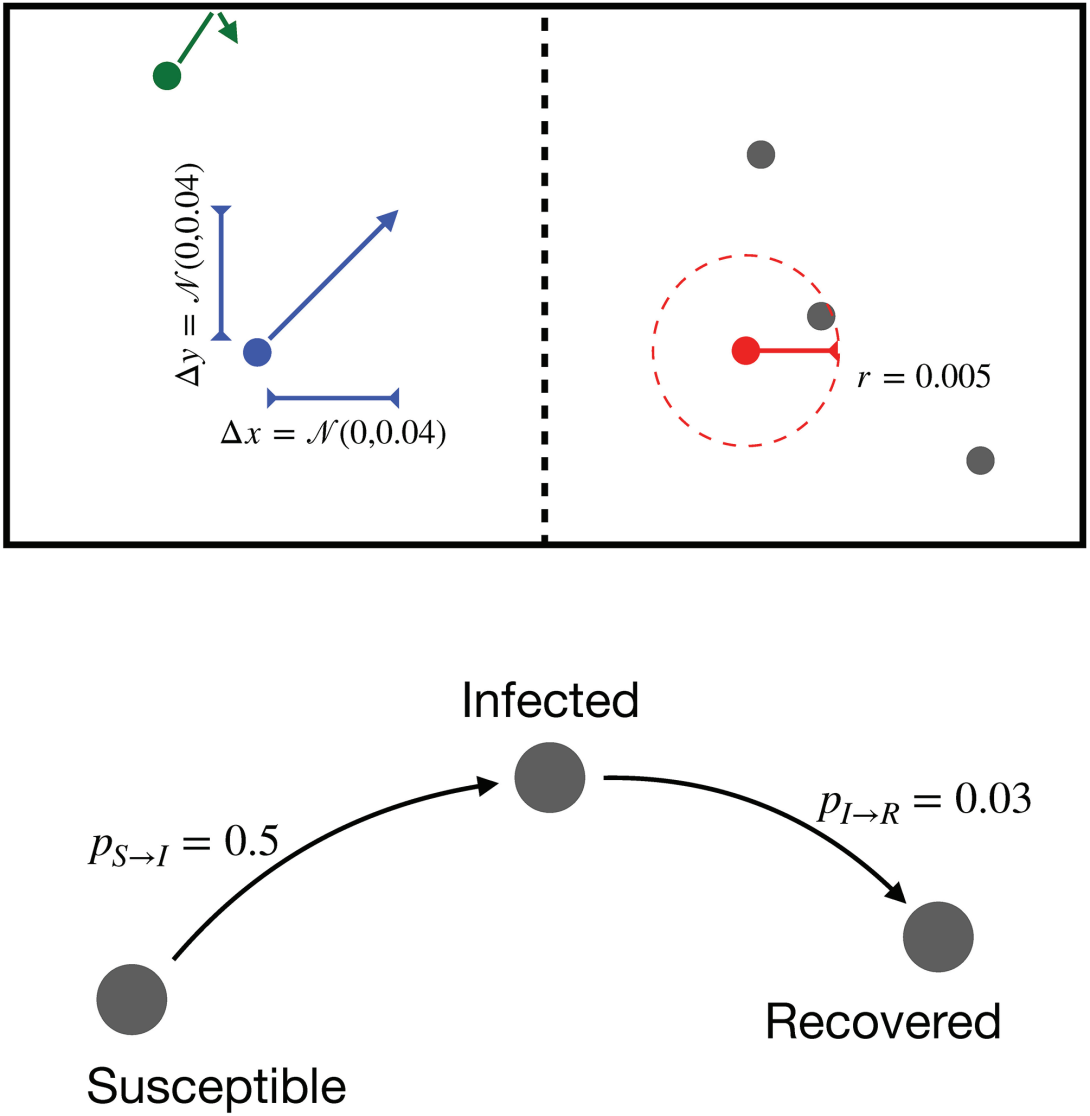
Initial propagation model. The top part depicts a spatial representation of the system, composed of two 1×1 boxes separated by an impermeable border (vertical dashed line). Also represented are the amplitude of the random walk of a person (blue), the bounce when encountering the external boundaries (green), and the radius of the infection (red); note that, for the sake of clarity, these magnitudes are not represented at scale. The bottom part depicts the compartments and transition probabilities of the SIR model.

The propagation of a generic contagious disease is described through a simple spatial model with mechanistic people movements. As depicted in the left of Fig. 1, we consider two contiguous boxes of size 1×1. A set of point-like agents, representing people, move inside them according to an uncorrelated random walk in which the change in both coordinates is given by a normal distribution N(0.0,0.04). When one of these people reaches the boundary of the box, it is reflected back. When compared to mass-action models, the model here used has the advantage of not assuming homogeneous mixing of susceptible and infectious people and of explicitly including spatial proximity as a key element of the dynamics^25^—an element needed to account for different people densities. Additionally, we initially consider the two boxes as independent, i.e., the border between both boxes (the vertical dashed line in Fig. 1) is impermeable and agents cannot move between them.

The dynamics of each individual is then described by a classical SIR model^23,24,26^ such that people are divided into three groups: *S*, susceptible, when they are healthy but can contract the disease; *I*, infected, when they are carrying the disease; and *R*, recovered, when they have passed the disease, are no longer contagious, and have developed an immunity. At the beginning of each simulation, 5% of the people are randomly chosen and set in the infected category. Afterward, people are allowed to freely move; when two people are within a range r, if one is in group *I* and the other in group *S*, the latter gets infected with probability $p_{S\to I}$. Additionally, infected people recover with probability $p_{I\to R}$. In what follows, these parameters are set to r=0.005, $p_{S\to I}=0.5$, and $p_{I\to R}=0.03$.

**FIG. 2.**
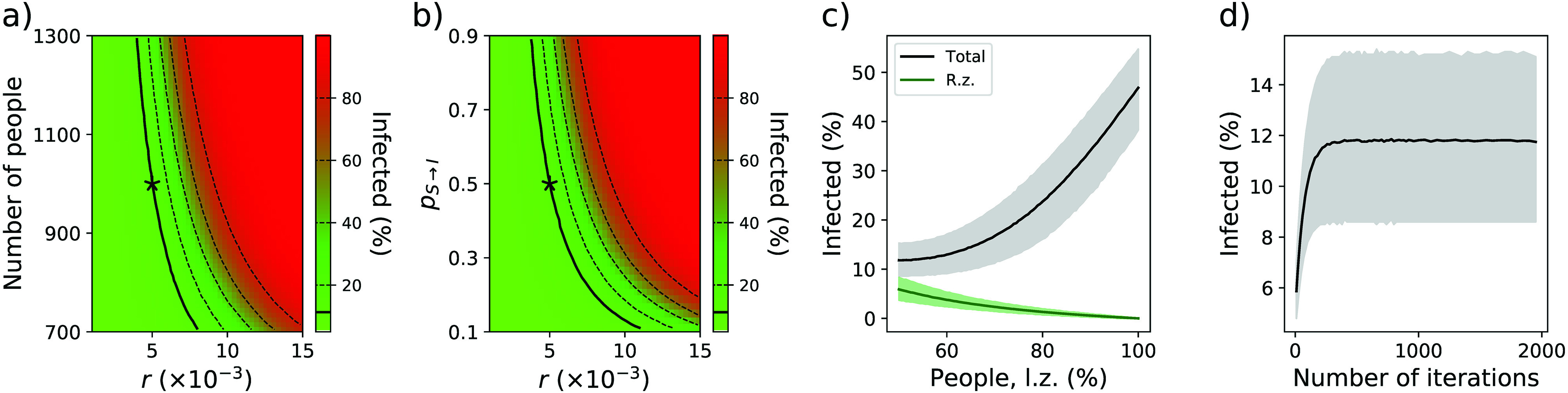
Results of the initial propagation model. (a) Evolution of the percentage of infected population as a function of r and the number of people in the system. (b) Evolution of the percentage of infected population as a function of r and the infection probability $p_{S\to I}$. Black stars indicate the parameter values used in this work, and solid black lines indicate the contour lines passing through those parameter values. (c) Evolution of the percentage of infected people (black line) and of the percentage of infected people in the right zone (green line) as a function of the percentage of people in the left zone. lz, left zone; rz, right zone. Black lines and gray bands, respectively, indicate the mean and 10–90 percentile over $10^{4}$ simulations. (d) Evolution of the percentage of infected population as a function of the number of iterations of the simulation.

This model is able to recover some basic results of epidemic dynamics, independently of the values assigned to its parameters. For instance, panel (a) of Fig. 2 shows the evolution of the percentage of infected individuals as a function of the population size and of r—a random 5% of individuals are initially infected. A non-linear behavior can be appreciated in which higher population densities foster disease propagation in a supralinear way. This same behavior is obtained independently of r, and the same number of infected people can be recovered, provided the other parameters, as, e.g., the total number of people [panel (a)] and $p_{S\to I}$ [panel (b)], are changed accordingly. The effect of the previously described non-linearity can be appreciated in panel (c) of Fig. 2, which reports the percentage of infected people (black line) and the percentage of infected people in the right zone (green line) as a function of the percentage of people in the left zone. While a uniform distribution between the two zones would result in a low contagion rate (of ≈12% of the total population), any asymmetry fosters propagation until the point at which half of the population gets infected if all individuals are concentrated in a single region. Finally, panel (d) of Fig. 2 reports the evolution of the percentage of infected people as a function of the number of iterations in the simulation (for $10^{3}$ people evenly distributed between the two zones); in order to ensure a steady-state solution, $10^{3}$ iterations are here used.

## III. RELAXING TRAVEL RESTRICTIONS

We next relax travel restrictions by loosening the isolation between the left and right zone, such that an individual reaching the middle barrier can cross it (instead of bouncing back) with a probability $p_{gate}$. To simplify the analysis, we consider that the left zone is the more densely populated region, and hence the one with the higher density of cases; people will thus only try to move from the left to the right zone. Also, the time scale of the SIR model is faster than the mobility one, such that a steady number of infected people is reached before all people can change zone.

**FIG. 3.**
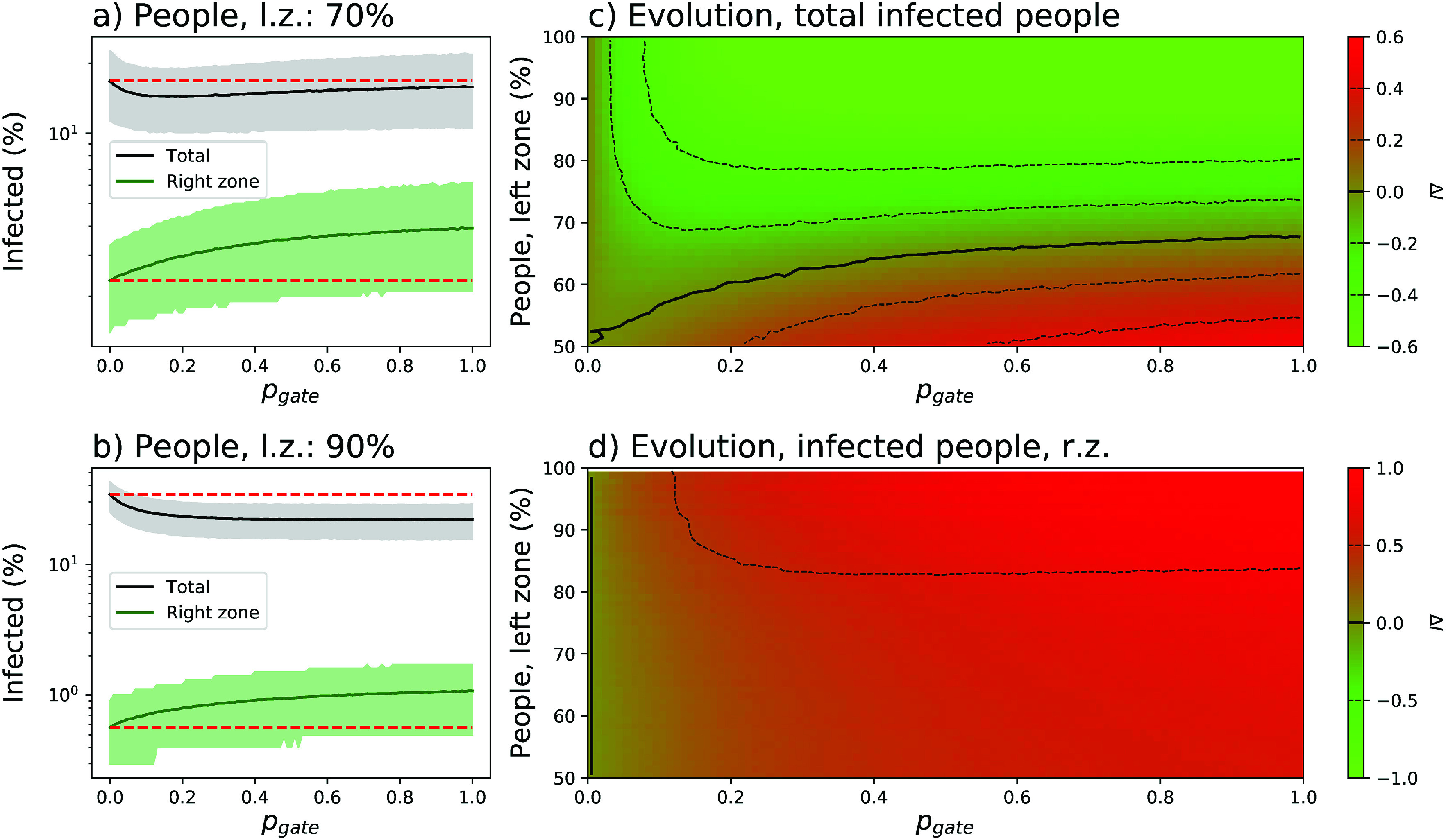
Relaxing travel restrictions. (a) and (b) Evolution of the percentage of infected population (black solid lines), and of infected population in the right zone (green solid lines), as a function of the permeability of the central gate ($p_{gate}$) and of the percentage of people initially in the left zone—70% for panel (a), and 90% for panel (b). lz, left zone; rz, right zone. Solid lines and bands respectively indicate the mean and 10–90 percentile over $10^{4}$ simulations. The horizontal red dashed lines indicate the result for $p_{gate}=0$. (c) and (d) Phase diagrams of the evolution of the total number of infected people (top), and of the number of infected people in the right zone (bottom), as a function of $p_{gate}$ and of the percentage of people in the left zone. Green and red shades indicate respectively a reduction and an increase in the number of infected people.

Panels (a) and (b) of Fig. 3 report the evolution of the percentage of infected people, both total and in the right zone, as a function of $p_{gate}$ (x axis) and of the asymmetry in the population distribution (70%, top panel, and 90%, bottom panel). It can be appreciated that allowing a reduced number of people to move from the left to the right zone implies a major overall benefit, especially for high asymmetries in the population distribution. Specifically, when 90% of the population is in the left box, allowing free movements to the right one (i.e., $p_{gate}=1.0$) reduces the fraction of infected people from a ≈35% to a ≈23%. This comes at a cost, and specifically an increase in the infection probability for people that were initially in the right zone. In the worst case, i.e., an asymmetry of 90% and $p_{gate}=1.0$, the percentage of people initially residing in the right zone and becoming infected almost doubles.

This behavior is further represented in panels (c) and (d) of Fig. 3, respectively, reporting the evolution of the total number of infected people and the number of infected people in the right zone as a function of both parameters. The plotted metric, ΔI, is defined as the base-2 logarithm of the relationship between the number of cases corresponding to the chosen parameters and the number of cases when $p_{gate}=0.0$. A value of +1 (respectively, −1) thus indicates doubling (halving) of the number of cases.

**FIG. 4.**
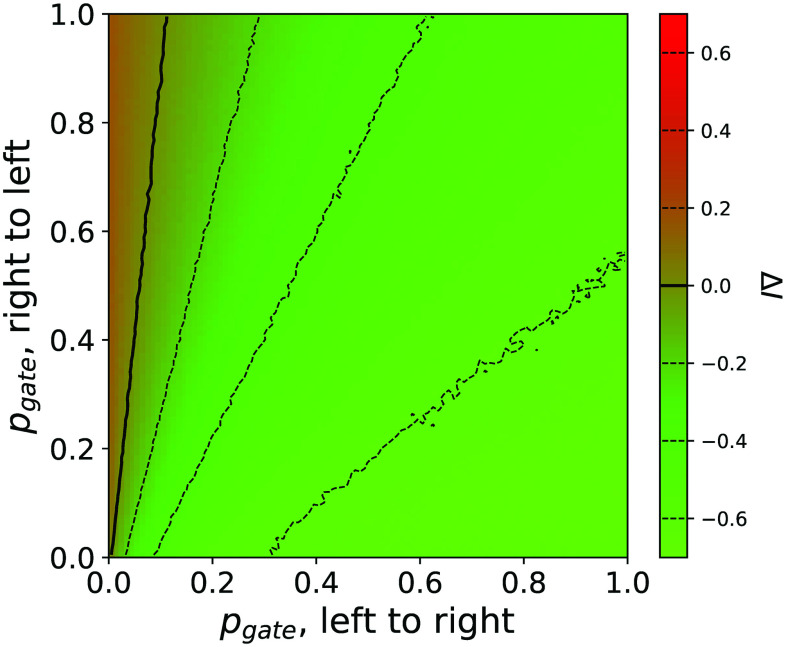
Bidirectional travels. The phase diagram of the evolution of the total number of infected people as a function of $p_{gate}$ for both left to right (x axis) and right to left (y axis) movements. 90% of the population is initially located in the right zone. Green and red shades indicate, respectively, a reduction and an increase in the number of infected people.

Due to the supralinear increase in the number of infected individuals as a function of the population density [see panels (a) and (c) of Fig. 2], any movement from the left to the right zone reduces the propagation in the former more than it increases it in the latter. A global reduction in the number of infected people is thus observed corresponding to the sum of a large negative contribution from the left zone and a small positive contribution from the right one. On the other hand, this positive effect is partly lost if we allow people to come back to the original region or more generally if we also allow right-to-left movements—see the phase diagram of Fig. 4. The unidirectionality of the mobility, and not generalized travel restrictions, is thus the essential ingredient to limit the spreading process. Additionally, as this beneficial effect is the result of the non-linearity of the relationship between people density and the final number of infected people, it is independent on the value of other parameters, such as, e.g., r or $p_{S\to I}$.

We further consider the possibility of executing tests to people trying to cross the border separating the two regions. Following what is common practice at some international airports, people who test negative to the disease are allowed to pass to the right region; on the other hand, those with a positive result are sent back—in this case, bounce back to the left region. We further suppose that the test has a false negative rate of $p_{test}$ but zero false positive rate. This latter hypothesis is suggested by two considerations. First, considering a non-zero probability of false positives is equivalent to a decrease in the value of $p_{gate}$; i.e., some people’s movements are blocked by chance. Second, modern polymerase chain reaction (PCR) and digital PCR (dPCR) tests, as the one currently used for COVID-19, have a negligible false positive rate but a significant rate of false negatives.^27,28^

**FIG. 5.**
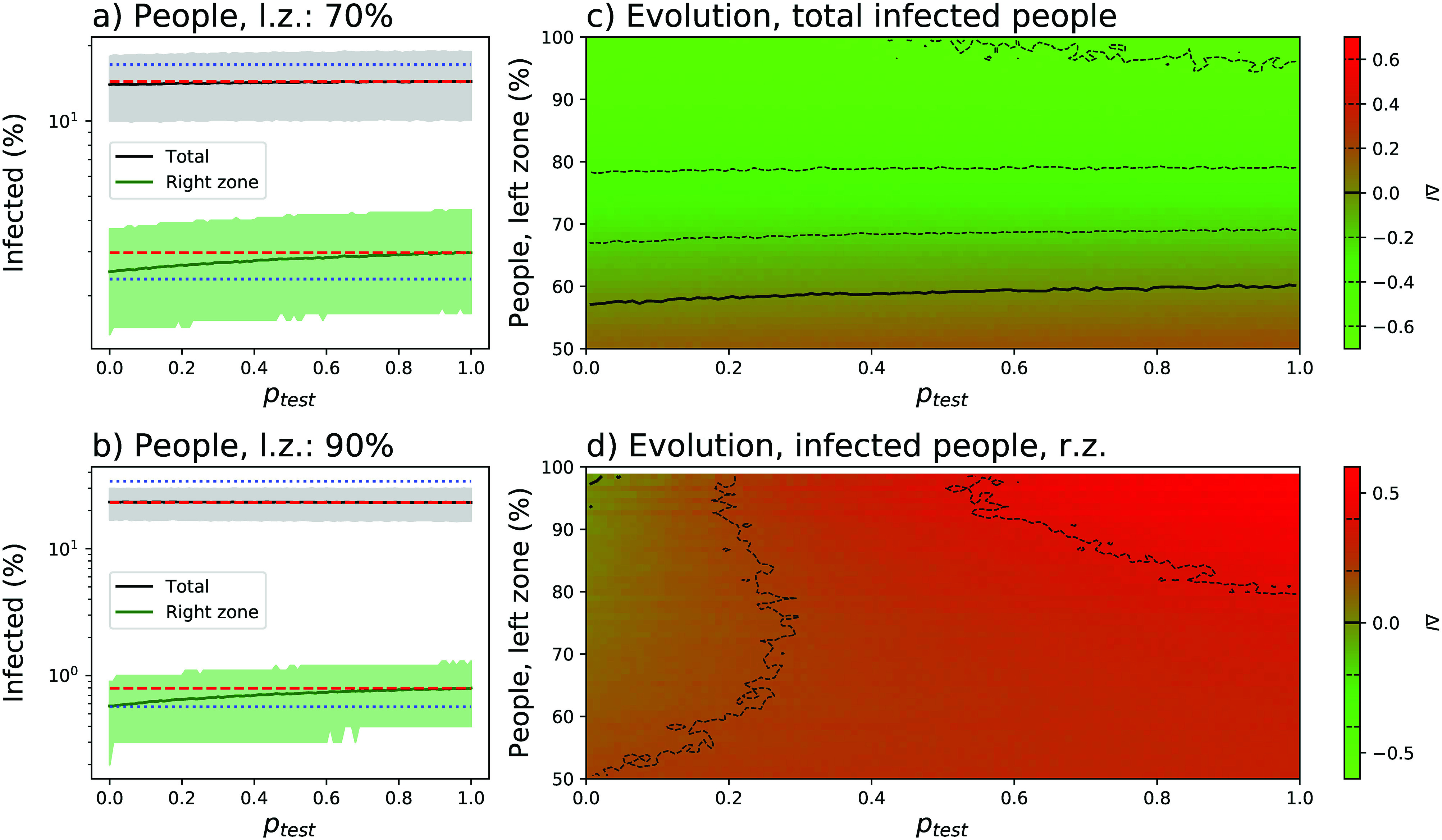
Applying tests to travelers. (a) and (b) Evolution of the percentage of the infected population (black solid lines) and of the infected population in the right zone (green solid lines) as a function of the rate of false negative of the test ($p_{test}$) and of the percentage of people initially in the left zone—70% for panel (a) and 90% for panel (b). lz, left zone; rz, right zone. Black lines and gray bands, respectively, indicate the mean and 10–90 percentile over $10^{4}$ simulations. The horizontal red dashed lines indicate the result for $p_{test}=1$, equivalent to when no test is actually performed; blue dotted lines indicate the results for $p_{gate}=0$, i.e., when the two regions are completely isolated. (c) and (d) Phase diagrams of the evolution of the total number of infected people (top) and of the number of infected people in the right zone (bottom) as a function of $p_{test}$ and of the percentage of people in the left zone. Green and red shades indicate, respectively, a reduction and an increase in the number of infected people.

As can be appreciated in Fig. 5, a small false negative rate (i.e., $p_{test}\approx 0$) does not significantly change the global infection rate, and values are similar to those obtained when no test is executed—see the red dashed horizontal lines in panels (a) and (b), which also correspond to the case in which $p_{test}\approx 1$; i.e., everyone gets a negative outcome. At the same time, the percentage of people infected in the right zone is reduced to almost the value observed in the isolated case (the latter represented by blue dotted horizontal lines). In other words, allowing only healthy people to move to the low-density region both improves the global situation and at the same time minimizes the impact in the low-density zone. Most notably, this latter impact changes smoothly with $p_{test}$ such that even tests with low precision can yield a significant benefit.

**FIG. 6.**
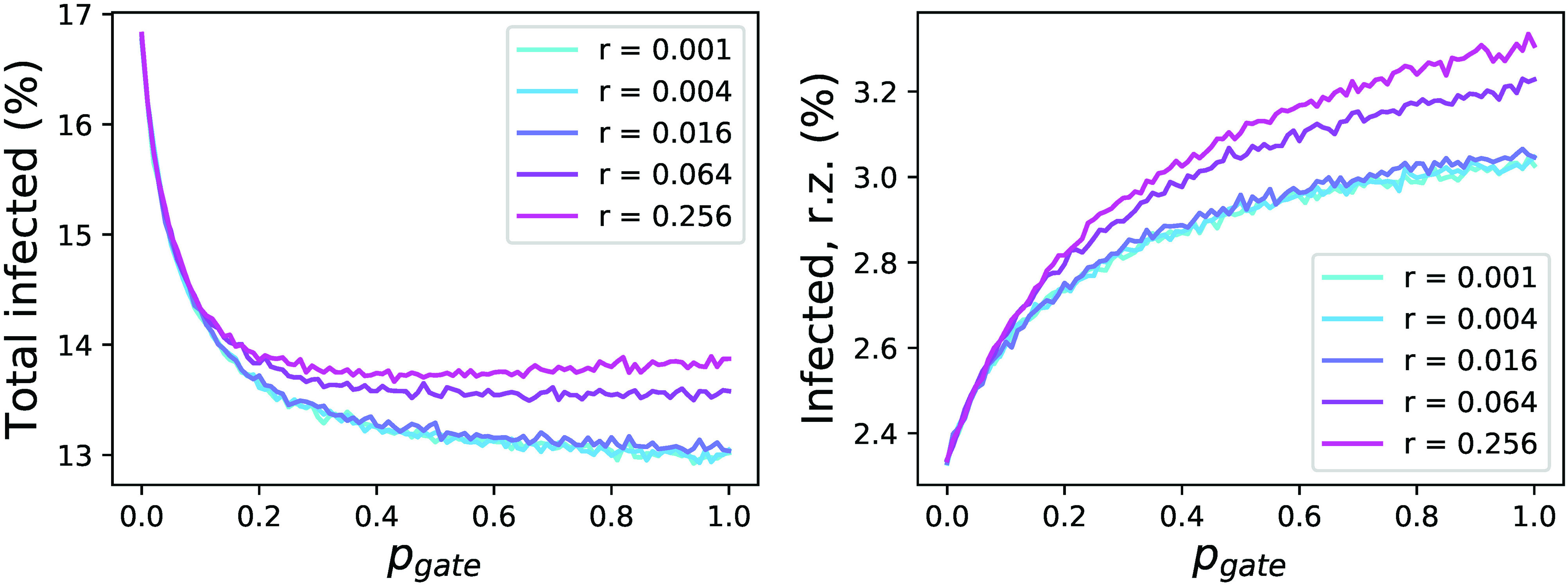
Imposing self-isolation to travelers. Left and right panels depict the evolution of, respectively, the percentage of the infected population and of the infected population in the right zone as a function of $p_{gate}$ and the radius of self-isolation r. rz, right zone.

We finally analyze the influence of a limited self-isolation policy. People who try to move from the left to the right region are not tested but are instead requested to self-isolate to avoid further propagations. This is simulated by choosing a position at random in the right region, by locating the person in that position after crossing the border, and by limiting the random movements of the person in a given radius r. As can be seen in Fig. 6, small values of r benefit both the full system and the right zone, as the probability for an infected person to interact with others is substantially reduced.

## IV. MINIMIZING DISEASE SPREADING THROUGH FORCED RELOCATION

If allowing free movement from the high- to the low-density region is beneficial to the system, the next logical question is whether this could be enforced in a proactive way. Specifically, we start with an initial asymmetric population distribution. Before running the simulation, a population sample is randomly selected from the high-density region and tested; those having a negative result are then moved to the low-density region. The simulation is then run normally, with $p_{gate}=0$ and no self-isolation. Note that this is qualitatively different from the previous analyses, as here, the relocation is forced at the beginning of the spreading process and is thus not a continuous stochastic process.

**FIG. 7.**
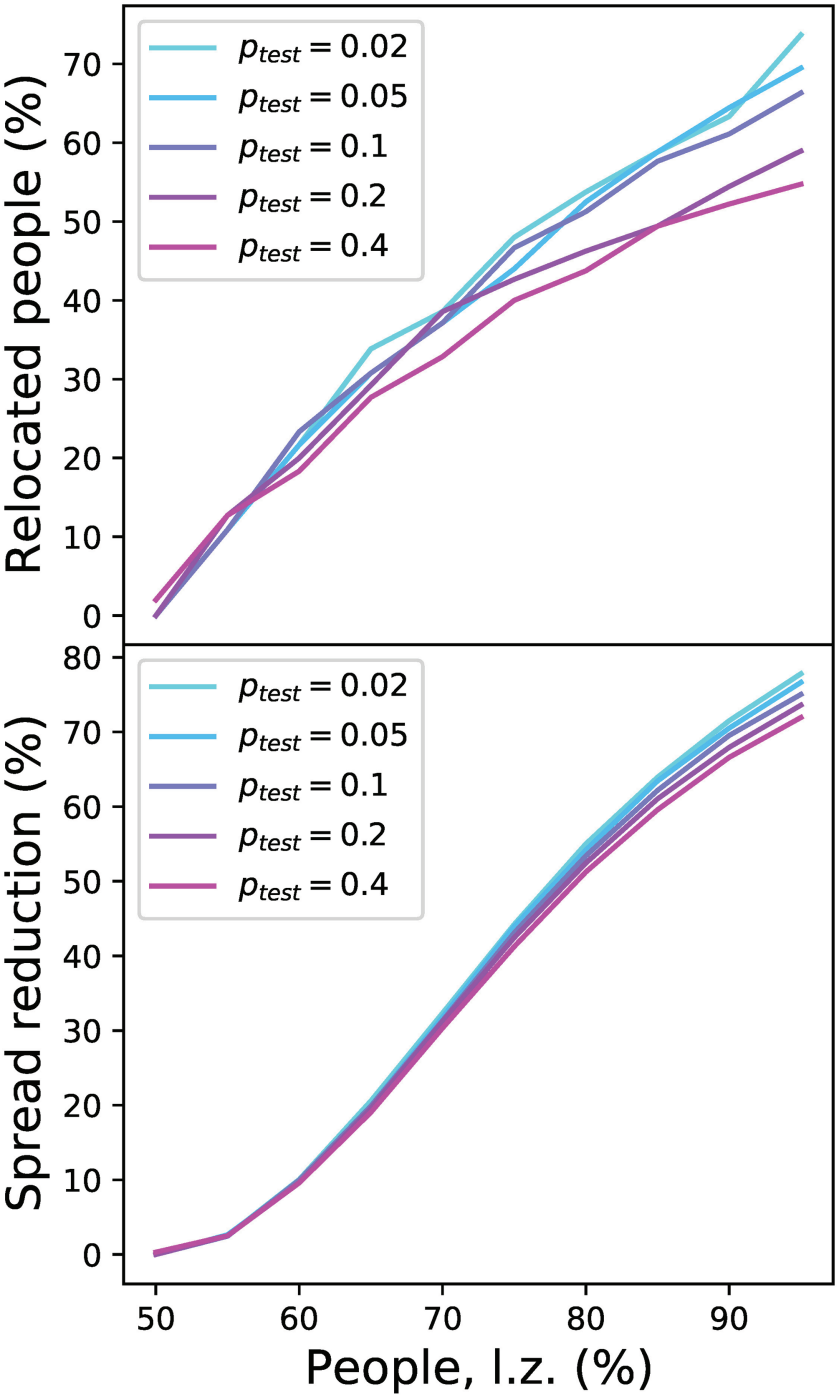
Forced relocation of people. Evolution of the optimal percentage of relocated people (top panel) and of the resulting reduction in the disease spread (bottom panel) as a function of the percentage of people in the left zone and of the rate of false negatives in the test ($p_{test}$). lz, left zone.

Figure 7 (top) reports the optimal percentage of the population that have to be relocated as a function of the initial asymmetry. Additionally, the bottom panel reports the reduction in the spreading of the disease, i.e., the reduction (in percentage) in the final number of cases. It can be appreciated that a large-scale forced relocation yields a substantial reduction in the number of cases. In other words, the best strategy entails moving a large quantity of healthy people to the (initially) low-density region, with the objective of effectively clustering patients in one region and healthy people in the other one. Notably, results are little sensitive to the rate of false negatives of the test ($p_{test}$, see the different lines), provided the number of relocated people is adjusted accordingly.

## V. DISCUSSION AND CONCLUSIONS

Travel restrictions are customarily used as a nonpharmaceutical measure to limit the propagation of contagious diseases, especially with the idea of preventing their attaining disease-free territories. The effectiveness of such a measure has nevertheless been challenged through theoretical and numerical arguments, and such an isolation can further have a huge negative economic impact.

As previously shown,^17–20^ when the dynamics of the disease (i.e., its effective reproduction number R) is driven by the density of people in different regions, with all other conditions being equal, allowing some degree of mixing is beneficial for the system as a whole. We here build on top of these results and focus on a specific mobility in which people move from high- to low-density regions—simulating what is really observed in, e.g., Spain during the COVID-19 pandemic.^21,22^ Due to the non-linear behavior of R as a function of the people density, such mobility results in an overall lower number of infections. This comes at the cost of a higher number of infections in the low-density region, which is effectively importing cases. Still, this drawback can substantially be reduced with policies such as tests at the border, even if the test has a significant rate of false negatives, and by reducing the mobility of relocated people. Thus, for instance, a sensible policy would be to allow people living in dense cities to move to a second house in the country-side, provided they agree to minimize social contacts there. The key to obtaining a positive effect is the unidirectionality of the mobility; i.e., people should not be allowed to move to the high-density region until the pandemic is over—a topic not previously studied.^17–20^ As shown in Fig. 7, an even more drastic solution could be designed, involving the forced relocation of healthy people at the beginning of the spreading process. While this allows for a drastic reduction in the number of infections, its real-world applicability is questionable at best.

As any other simplified model, it is worth noting that the one here presented has several limitations. First of all, this model is based on the hypothesis that the only factor influencing the contagion rate of the disease is the population density. Denser populations imply a higher probability for two people to cross their path or interact and hence a faster propagation. Most importantly, other elements that may affect the propagation are not taken into account, such as cultural and environmental factors. We also suppose the same virus strand in all regions; thus, there is no risk of importing different (and maybe more aggressive) strands.

Second, this model does not take into account the differential pressure on regional healthcare systems. Healthcare infrastructures are usually sized according to the population they service. Hence, moving cases from high to low density areas may result in too high a pressure for the healthcare system in the latter zones and eventually in its saturation.^29,30^

Finally, there are also political and ethical concerns. On one hand, politicians in a given region may feel that their responsibility is toward the people living there; they may thus prefer to protect their voters, as opposed to looking for the global interest. On the other hand, the mobility strategy here discussed implies incrementing the number of cases in regions that were not strongly affected by the disease; these regions have thus to accept a worsening in their situation in exchange for a global benefit, something, in general, difficult to digest.

In spite of these limitations, the work here proposed suggests a new venue for research and a provocative thought about a policy that is customarily applied, but whose effect could even be globally negative.

## Data Availability

The data that support the findings of this study are available within the article.

## References

[1] D. Defoe, *A Journal of the Plague Year* (E. Nutt, 1722).

[2] T. P. Velavan and C. G. Meyer, “The COVID-19 epidemic,” Trop. Med. Int. Health 25, 278 (2020). 10.1111/tmi.13383 32052514 PMC7169770

[3] M. Chinazzi, J. T. Davis, M. Ajelli, C. Gioannini, M. Litvinova, S. Merler, A. P. Y. Piontti, K. Mu, L. Rossi, K. Sun *et al.*, “The effect of travel restrictions on the spread of the 2019 novel coronavirus (COVID-19) outbreak,” Science 368, 395–400 (2020). 10.1126/science.aba9757 32144116 PMC7164386

[4] L. O. Gostin and L. F. Wiley, “Governmental public health powers during the COVID-19 pandemic: Stay-at-home orders, business closures, and travel restrictions,” JAMA 323, 2137–2138 (2020). 10.1001/jama.2020.5460 32239184

[5] S. Devi, “Travel restrictions hampering COVID-19 response,” Lancet 395, 1331–1332 (2020). 10.1016/S0140-6736(20)30967-3 32334692 PMC7180010

[6] D. M. Studdert, M. A. Hall, and M. M. Mello, “Partitioning the curve—Interstate travel restrictions during the COVID-19 pandemic,” New Engl. J. Med. 383, e83 (2020). 10.1056/NEJMp2024274 32757517

[7] K. Linka, M. Peirlinck, F. Sahli Costabal, and E. Kuhl, “Outbreak dynamics of COVID-19 in Europe and the effect of travel restrictions,” Comput. Methods Biomech. Biomed. Eng. 23, 710–717 (2020). 10.1080/10255842.2020.1759560 PMC7429245 32367739

[8] M. Mazzoli, D. Mateo, A. Hernando, S. Meloni, and J. J. Ramasco, “Effects of mobility and multi-seeding on the propagation of the COVID-19 in Spain,” medRxiv (2020).

[9] S. Flaxman, S. Mishra, A. Gandy, H. J. T. Unwin, T. A. Mellan, H. Coupland, C. Whittaker, H. Zhu, T. Berah, J. W. Eaton *et al.*, “Estimating the effects of non-pharmaceutical interventions on COVID-19 in Europe,” Nature 584, 257–261 (2020). 10.1038/s41586-020-2405-7 32512579

[10] S. Hsiang, D. Allen, S. Annan-Phan, K. Bell, I. Bolliger, T. Chong, H. Druckenmiller, L. Y. Huang, A. Hultgren, E. Krasovich *et al.*, “The effect of large-scale anti-contagion policies on the COVID-19 pandemic,” Nature 584, 262–267 (2020). 10.1038/s41586-020-2404-8 32512578

[11] M. Zanin and D. Papo, “Assessing functional propagation patterns in COVID-19,” Chaos Solitons Fractals 138, 109993 (2020). 10.1016/j.chaos.2020.109993 32546901 PMC7290208

[12] T. D. Hollingsworth, N. M. Ferguson, and R. M. Anderson, “Will travel restrictions control the international spread of pandemic influenza?,” Nat. Med. 12, 497–499 (2006). 10.1038/nm0506-497 16675989

[13] J. M. Epstein, D. M. Goedecke, F. Yu, R. J. Morris, D. K. Wagener, and G. V. Bobashev, “Controlling pandemic flu: The value of international air travel restrictions,” PLoS One 2, e401 (2007). 10.1371/journal.pone.0000401 17476323 PMC1855004

[14] P. Bajardi, C. Poletto, J. J. Ramasco, M. Tizzoni, V. Colizza, and A. Vespignani, “Human mobility networks, travel restrictions, and the global spread of 2009 H1N1 pandemic,” PLoS One 6, e16591 (2011). 10.1371/journal.pone.0016591 21304943 PMC3031602

[15] S. Ryu, H. Gao, J. Y. Wong, E. Y. Shiu, J. Xiao, M. W. Fong, and B. J. Cowling, “Nonpharmaceutical measures for pandemic influenza in nonhealthcare settings—International travel-related measures,” Emerg. Infect. Dis. 26, 961 (2020). 10.3201/eid2605.190993 32027587 PMC7181936

[16] M. Eichner, M. Schwehm, N. Wilson, and M. G. Baker, “Small islands and pandemic influenza: Potential benefits and limitations of travel volume reduction as a border control measure,” BMC Infect. Dis. 9, 160 (2009). 10.1186/1471-2334-9-160 19788751 PMC2761921

[17] J. Gómez-Gardenes, D. Soriano-Panos, and A. Arenas, “Critical regimes driven by recurrent mobility patterns of reaction–diffusion processes in networks,” Nat. Phys. 14, 391–395 (2018). 10.1038/s41567-017-0022-7

[18] D. Soriano-Paños, L. Lotero, A. Arenas, and J. Gómez-Gardeñes, “Spreading processes in multiplex metapopulations containing different mobility networks,” Phys. Rev. X 8, 031039 (2018). 10.1103/PhysRevX.8.031039

[19] D. Soriano-Panos, G. Ghoshal, A. Arenas, and J. Gómez-Gardenes, “Impact of temporal scales and recurrent mobility patterns on the unfolding of epidemics,” J. Stat. Mech.: Theory Exp. 2020, 024006). 10.1088/1742-5468/ab6a04

[20] D. Soriano-Paños, J. H. Arias-Castro, A. Reyna-Lara, H. J. Martínez, S. Meloni, and J. Gómez-Gardeñes, “Vector-borne epidemics driven by human mobility,” Phys. Rev. Res. 2, 013312 (2020). 10.1103/PhysRevResearch.2.013312

[21] RT, see https://www.rt.com/news/502629-madrid-criticized-ignoring-covid-restrictions/ for “‘Out in Their Masses’: Madrid Residents Criticized for Ignoring New Covid Restrictions as Spain Sees Jump in Cases” (accessed October 6, 2020).

[22] El País, see https://english.elpais.com/economy_and_business/2020-0917/fearing-new-coronavirus-lockdown-spaniards-look-for-bigger-homes-outside-of-the-city.html for “Fearing New Coronavirus Lockdown, Spaniards Look for Bigger Homes Outside of the City” (accessed October 6, 2020).

[23] W. O. Kermack and A. G. McKendrick, “A contribution to the mathematical theory of epidemics,” Proc. R. Soc. Lond. Ser. A 115, 700–721 (1927). 10.1098/rspa.1927.0118

[24] H. W. Hethcote, “The mathematics of infectious diseases,” SIAM Rev. 42, 599–653 (2000). 10.1137/S0036144500371907

[25] A. M. Fofana and A. Hurford, “Mechanistic movement models to understand epidemic spread,” Philos. Trans. R. Soc. B 372, 20160086 (2017). 10.1098/rstb.2016.0086 PMC5352813 28289254

[26] R. Pastor-Satorras, C. Castellano, P. Van Mieghem, and A. Vespignani, “Epidemic processes in complex networks,” Rev. Mod. Phys. 87, 925 (2015). 10.1103/RevModPhys.87.925

[27] D. Hospodsky, N. Yamamoto, and J. Peccia, “Accuracy, precision, and method detection limits of quantitative PCR for airborne bacteria and fungi,” Appl. Environ. Microbiol. 76, 7004–7012 (2010). 10.1128/AEM.01240-10 20817798 PMC2976253

[28] R. Lu, J. Wang, M. Li, Y. Wang, J. Dong, and W. Cai, “SARS-CoV-2 detection using digital PCR for COVID-19 diagnosis, treatment monitoring and criteria for discharge,” medRxiv (2020).

[29] C. A. Favero, see https://ssrn.com/abstract=3566865 for “Why Is COVID-19 Mortality in Lombardy So High? Evidence from the Simulation of a SEIHCR Model” (April 2, 2020).

[30] D. Papo, M. Righetti, L. Fadiga, F. Biscarini, and M. Zanin, “A minimal model of hospital patients’ dynamics in COVID-19,” Chaos Solitons Fractals 140, 110157 (2020). 10.1016/j.chaos.2020.110157 32834645 PMC7386477

